# Early physical rehabilitation dosage in the intensive care unit associates with hospital outcomes after critical COVID-19

**DOI:** 10.1186/s13054-024-05035-6

**Published:** 2024-07-18

**Authors:** Kirby P. Mayer, Evan Haezebrouck, Lori M. Ginoza, Clarisa Martinez, Minnie Jan, Lori A. Michener, Lindsey E. Fresenko, Ashley A. Montgomery-Yates, Anna G. Kalema, Amy M. Pastva, Michelle Biehl, Matthew F. Mart, Joshua K. Johnson

**Affiliations:** 1 Department of Physical Therapy, College of Health Sciences, University of Kentucky, 900 Rose Street, Lexington, KY 40536 USA; 2 University of Michigan Hospital, University of Michigan Health, Ann Arbor, MI USA; 3 Division of Biokinesiology and Physical Therapy, University of Southern California, Los Angeles, CA USA; 4 Department of Internal Medicine, College of Medicine, University of Kentucky, Lexington, KY USA; 5 Division of Physical Therapy, Duke University School of Medicine, Durham, NC USA; 6 Department of Physical Medicine and Rehabilitation, Cleveland Clinic, Cleveland, OH USA; 7 Division of Allergy, Pulmonary, and Critical Care, Vanderbilt University Medical Center, Nashville, TN USA; 8 Critical Illness, Brain Dysfunction, and Survivorship (CIBS) Center, Nashville, TN USA; 9 Geriatric Research, Education, and Clinical Center (GRECC), Tennessee Valley Veterans Affairs Healthcare System, Nashville, TN USA

**Keywords:** Physical rehabilitation, Early mobilization, Critical illness, Occupational therapy, Physical therapy, COVID

## Abstract

**Objective:**

To examine the relationship between physical rehabilitation parameters including an approach to quantifying dosage with hospital outcomes for patients with critical COVID-19.

**Design:**

Retrospective practice analysis from March 5, 2020, to April 15, 2021.

**Setting:**

Intensive care units (ICU) at four medical institutions.

**Patients:**

n = 3780 adults with ICU admission and diagnosis of COVID-19.

**Interventions:**

We measured the physical rehabilitation treatment delivered in ICU and patient outcomes: (1) mortality; (2) discharge disposition; and (3) physical function at hospital discharge measured by the Activity Measure-Post Acute Care (AM-PAC) “6-Clicks” (6–24, 24 = greater functional independence). Physical rehabilitation dosage was defined as the average mobility level scores in the first three sessions (a surrogate measure of intensity) multiplied by the rehabilitation frequency (PT + OT frequency in hospital).

**Measurements and main results:**

The cohort was a mean 64 ± 16 years old, 41% female, mean BMI of 32 ± 9 kg/m^2^ and 46% (n = 1739) required mechanical ventilation. For 2191 patients who received rehabilitation, the dosage and AM-PAC at discharge were moderately, positively associated (Spearman’s rho [r] = 0.484, *p* < 0.001). Multivariate linear regression (model adjusted R^2^ = 0.68, *p* < 0.001) demonstrates mechanical ventilation (β = − 0.86, *p* = 0.001), average mobility score in first three sessions (β = 2.6, *p* < 0.001) and physical rehabilitation dosage (β = 0.22, *p* = 0.001) were predictive of AM-PAC scores at discharge when controlling for age, sex, BMI, and ICU LOS.

**Conclusions:**

Greater physical rehabilitation exposure early in the ICU is associated with better physical function at hospital discharge.

## Brief report

### Objectives

Exercise and early mobility are key components of clinical practice guidelines for patients with critical illness, as defined in the Intensive Care Unit (ICU) Liberation bundle [1]. However, findings from multiple randomized ICU rehabilitation trials have been equivocal, demonstrating minimal impact on mortality and physical function [2–5]. A potential explanation for the lack of benefits is a non-specific exercise dose. Patients are routinely randomized to “one-size-fits-all” protocols leading to heterogeneity in the response to treatment. Dosage that accounts for the frequency and intensity of exercise is frequently overlooked or not addressed in critical care practice and research. The rehabilitation dosage delivered in large randomized controlled trials (RCTs) is rarely implemented in clinical practice [6, 7], and patients seldomly receive a targeted or individualized dose of exercise. Patients with critical COVID-19 have not been studied to determine if dosage of exercise is related to outcomes. The COVID-19 pandemic may have unintentionally altered patterns in rehabilitation practice due to periods of isolation [8]. Thus, the primary objective of this study was to examine the relationship between physical rehabilitation parameters including an approach to quantifying dosage with hospital outcomes for patients with critical COVID-19.

### Design

Retrospective practice analysis for patients hospitalized from March 5, 2020, to April 15, 2021.

### Setting

ICUs at four academic medical institutions (University of Kentucky, Cleveland Clinic, University of Michigan, and University of Southern California).

### Patients

3780 adults (≥ 18 years of age) admitted to ICU with primary diagnosis of COVID-19.

### Interventions

We examined the relationship between ICU-based physical rehabilitation interventions and hospital-based outcomes. Outcomes included: (1) mortality; (2) discharge disposition; and 3) physical function at or near hospital discharge measured by the Activity Measure-Post Acute Care (AM-PAC) “6-Clicks” Inpatient Mobility Short Form (6–24, 24 = greater functional independence) [9]. Physical rehabilitation parameters included time to first rehabilitation (physical [PT] or occupational [OT]) session in days, number of PT and OT sessions completed during hospital length of stay (LOS), frequency of PT and OT (# of session/hospital LOS), mobility status during first three and the last recorded (if more than 3 sessions) rehabilitation sessions. Mobility levels were quantified by the John Hopkins-Highest Level of Mobility (JH-HLM, 1–8, 1 = lying in bed; 8 = ambulating > 250 feet). The physical rehabilitation dose was quantified as the average JH-HLM score over the first three sessions (a surrogate measure of early intensity) multiplied by the rehabilitation frequency (PT + OT frequency). The dose provides information on delivery of ICU rehabilitation such that patients who achieve high mobility with daily frequency of rehabilitation receive the highest dosage, whereas patients with lower mobility levels and infrequent rehabilitation receive the lowest dose. Our method is based on our previous published studies demonstrating that the mobility levels obtained in the first 3 rehabilitation sessions predict, or at minimum, associate with patient-centered outcomes [10, 11].

### Measurements and main results

Descriptive statistics were reported as mean ± SD, median [IQR], or n (%) as appropriate. A total of 3780 patients with COVID critical illness were included. Patients were stratified into groups according to discharge disposition (in-hospital death, subacute or long-term care facility, acute rehabilitation facility, home with services, or home independent). The change in mobility level during rehabilitation as measured by JH-HLM among discharge groups were compared using a two-way ANOVA. Dose of rehabilitation between discharge disposition groups were compared using Tukey’s multiple comparison test. Univariate analyses (Spearman’s correlation) were performed to assess associations between rehabilitation parameters and functional outcomes. Multivariate linear regression was performed to analyze the association between rehabilitation dose and discharge AM-PAC scores, which defined physical function among survivors, adjusting for pre-specified covariates including age, sex, body mass index (BMI), ICU length of stay, and receipt of mechanical ventilation.

Patient demographics are described in Table 1. The cohort was a mean 64 ± 16 years old, 41% female and mean BMI of 32 ± 9 kg/m^2^. Mechanical ventilation was required in 46% (n = 1739), and the median hospital LOS was 12 days (IQR 7–21). A total of 2200 (58%) and 1698 (45%) patients received at least one PT and OT session, respectively. The first rehabilitation session occurred 7.5 ± 8.0 days after ICU admission. Patients received PT at a frequency of 0.22 ± 0.14 days a week and OT at a frequency of 0.18 ± 0.11 days a week, equivalent to 2.8 rehabilitation sessions per week. Mobility levels on the JH-HLM scale generally increased from the first to last session (+ 0.93 ± 2.1). The mean JH-HLM score for all sessions was 4.6 ± 1.7; this suggests a likely ability to transfer from a bed to a chair but not stand for up to one minute. The mean dose of physical rehabilitation was 1.8 ± 1.3 units.

**Table 1:** Demographic, clinical and rehabilitation parameters

Parameter	In-hospital mortality (n = 994)	Survivor (n = 2788)	*p* value
Age, years, mean (SD)	70.8 ± 13	62. 2 ± 16	< 0.001
Sex			0.687
Female, n (%)	411 (41)	1142 (41)	
Male, n (%)	583 (59)	1644 (59)	
Race			0.670
White/Caucasian, n (%)	660 (66)	1724 (62)	
Black/African American, n (%)	258 (26)	859 (31)	
Asian, n (%)	6 (0.6)	17 (0.6)	
Unknown/did not disclose, n (%)	70 (7)	188 (7)	
Ethnicity			0.415
Hispanic, n (%)	75 (8)	194 (7)	
Non-Hispanic, n (%)	901 (91)	2525 (91)	
Unknown, n (%)	18 (1)	69 (2)	
BMI (kg/m^2^)	30.7 ± 8	32.4 ± 9	< 0.001
Mechanical Ventilation, yes, n (%)	725 (73)	1013 (36)	< 0.001
Mechanical Ventilation duration days, mean (SD)	12.4 ± 13	14.2 ± 15.4	0.011
RASS, median [IQR]	− 1.4 ± 1.7	− 0.6 ± 1.2	< 0.001
PT, yes, n (%)	354 (35)	1846 (66)	< 0.001
OT, yes, n (%)	275 (34)	1423 (57)	< 0.001
Time to 1st PT, days, mean (SD)	5.8 ± 7.8	7.8 ± 8.1	< 0.001
Time to 1st OT, days, mean (SD)	5.6 ± 6.9	7.8 ± 7.6	< 0.001
Number of total PT sessions, mean (SD)	2.8 ± 5.7	4.6 ± 5.2	< 0.001
Number of total OT sessions, mean (SD)	2.4 ± 3.4	3.1 ± 3.6	< 0.001
PT Frequency days / week	0.16 ± 0.11	0.24 ± 0.14	< 0.001
OT Frequency days / week	0.13 ± 0.10	0.18 ± 0.11	< 0.001
AM-PAC at PT evaluation (6–24, 24 = greater functional independence)	12.3 ± 5.3	14.2 ± 5.8	< 0.001
AM-PAC, last recorded (6–24, 24 = greater functional independence)	11.3 ± 5.1	16.4 ± 5.7	< 0.001
AMPAC Change	− 0.9 ± 3.3	2.2 ± 4.3	< 0.001
JHHLM-initial	3.9 ± 1.8	4.4 ± 1.8	< 0.001
JHHLM-2nd	3.5 ± 1.8	4.6 ± 1.9	< 0.001
JHHLM-3rd	3.5 ± 1.8	4.6 ± 1.9	< 0.001
JHHLM-4th	3.0 ± 1.4	4.9 ± 1.9	< 0.001
JHHLM change	− 0.8 ± 1.7	1.1 ± 2.0	< 0.001
Rehabilitation dosage (JHHLM average * Frequency)	0.97 ± 0.73	1.90 ± 1.4	< 0.001
ICU LOS, days, median [IQR]	11.6 ± 12.5	8.3 ± 11.2	< 0.001
Hospital LOS, days, median [IQR]	15.5 ± 13.6	16.6 ± 16.1	0.064

BMI, body mass index; RASS, Richmond Agitation Sedation Scale; PT, physical therapy; OT, occupational therapy; AMPAC, Activity Measure-Post Acute Care (AM-PAC) “6-Clicks” Inpatient Mobility Short Form; JHHLM, the John Hopkins-Highest Level of Mobility; LOS, length of stay

**Fig. 1:**
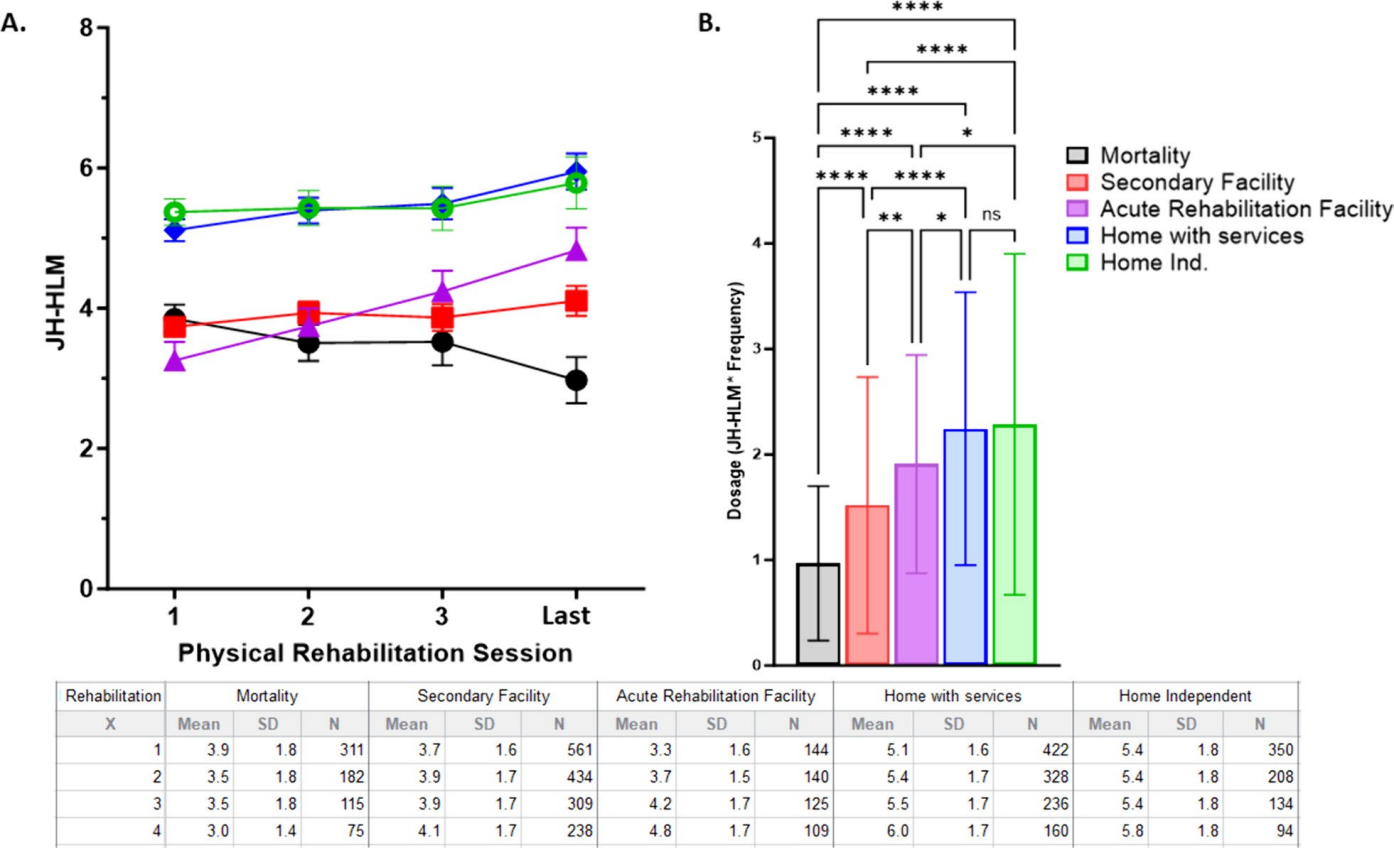
Dosage and change in mobility during physical rehabilitation grouped based on discharge disposition. **A** The change in mobility level measured by John Hopkins-Highest Level of Mobility (JH-HLM, 0–8) stratified by discharge disposition (black—mortality in hospital; red = secondary facilty; purple = acute rehabilitation facilty; blue = home with services; green = home without services). Two-Way ANOVA demonstrated significant difference in JH-HLM scores based on group (F = 240.8, *p* < 0.0001) and change over rehabilitation sessions (F = 11.13, *p* < 0.00) with interaction (F = 6.7, *p* < 0.0001). **B** The dosage of rehabilitation (JH-HLM average over four reported sessions multiplied by the frequency of rehabilitation) is significantly different based on discharge disposition (F = 69, *p* < 0.0001) with Tukey’s multiple comparison test revealing significant differences at the *p* < 0.0001 denoted with ****; *p* < 0.001 denoted with **, and * denoting *p* < 0.05. **C** (Table) provides raw data for the mobility sessions during the first 3 (1–3) sessions and the last rehabilitation (4) stratified based on the discharge destination

Patients who died in the hospital (n = 994, 26%) were older, more likely to require mechanical ventilation, had longer durations of mechanical ventilation, less likely to receive PT or OT, and had longer ICU LOS (Table 1) compared to patients who survived to hospital discharge. Compared to survivors, those who died in the hospital had an earlier start of rehabilitation, but had lower frequencies of rehabilitation, achieved lower levels of mobility, and received a lower dose of physical rehabilitation (Table 1). Stratified by discharge disposition, patients discharged to home had the highest dose of rehabilitation (F = 69, *p* < 0.0001; Fig. 1).

For 2191 patients who received PT or OT treatment and functional data were available in the EMR, the mean AM-PAC scores at discharge were 15.6 ± 5.9; suggesting a high-level of assistance is needed for bed-to-chair transfers [12]. Rehabilitation dose and physical function as measured by AM-PAC at discharge were moderately, positively associated (Spearman’s rho [r] = 0.484, *p* < 0.001). AM-PAC at discharge was also significantly associated with average mobility achieved in first 3 sessions (r = 0.799, *p* < 0.001), change in mobility from first to last session (r = 0.445, *p* < 0.001), and PT and OT frequency with physical function (r = 0.130, *p* < 0.001). Multivariate linear regression (model adjusted R^2^ = 0.68, *p* < 0.001) demonstrates the receipt of mechanical ventilation (β = − 0.86, *p* = 0.001), average mobility score in first three sessions (β = 2.6, *p* < 0.001) and physical rehabilitation dosage (β = 0.22, *p* = 0.001) were predictive of AM-PAC scores at discharge when controlling for age, sex, BMI, and ICU LOS.

## Conclusions

Our study, involving over 2000 critically ill patients with COVID-19 at four academic medical centers, underscores the pivotal role of physical rehabilitation exposure in the ICU, demonstrating a significant correlation with favorable hospital outcomes. These findings align with previous research highlighting the positive association between rehabilitation frequency and outcomes in COVID-19 patients, albeit not specifically focusing on those with critical illness [13]. We measured rehabilitation dose by assessing both session frequency and a surrogate marker of intensity derived from achieved mobility levels during sessions. Although our approach lacks physiologic dosage markers such as vital signs, timing of rehabilitation, and duration of intensity, it introduces a method for quantifying dose with a single unit. Adopting this approach may enable ICU rehabilitation programs to specifically delineate the intervention provided and anticipate patient benefit. In future, stratification and phenotypic analysis accounting for dose hold promise to guide interventions in clinical research settings.

Precision exercise, dose–response, and individual response heterogeneity are concepts in exercise dosing that are known to impact outcomes in older adults and diverse patient populations [14–16]. The intra-individual variations have also been recognized in critical care with ventilatory and pharmaceutical interventions [17, 18]. To improve the ICU rehabilitation field, it is imperative that clinicians and researchers examine the dose–response. As clinicians, we must modify and adjust treatments based on patient- and clinical-related factors to enhance outcomes. Our work as well as other research demonstrates that older individuals and patients with chronic disease respond differently to rehabilitation and exercise interventions [11, 19]. Thus, we strongly suggest that researchers and clinicians must begin to examine the response to targeted or individualized rehabilitation dose. The approach using mobility as a surrogate marker of intensity has limitation and may be further strengthen by including physiological response and timing.

Our study has several strengths including a large sample size from multiple academic medical centers with functioning ICU rehabilitation programs. We also used real world data directly from each rehabilitation session provided to create our method. It is important to highlight that a large percentage of patients did not receive or received a very low frequency of PT and OT in the ICU. Low delivery of mobilization and rehabilitation in the ICU remains common in clinical practice in ICU [6, 20] even despite clinical practice guidelines [1]. Disparate delivery of rehabilitation in practice as well as missing or limited functional mobility data in the EMR may have limited our findings. The retrospective design limits our ability to draw definitive conclusions with regard to causation and is at risk of residual confounding, so our findings should be considered hypothesis-generating. The study design may introduce representation and selection biases as it is impossible to control for all potential confounders, although, our modeling did account for receipt of MV and ICU length of stay. Still yet, patients who survived ICU may have had lower severity of illness and participated at higher levels of mobility with rehabilitation, regardless of the dose. Lastly, we could not account for duration of exercise intensity in our models; longer physiologic demands may produce additional benefits which warrants future investigations. In conclusion, we found that greater dose of rehabilitation during critical illness due to COVID-19 was associated with improved outcomes. Future studies should utilize personalized rehabilitation doses and identify the most optimal personalized dosage of rehabilitation in critically ill patients, including those with COVID-19.

## Data Availability

The datasets generated and/or analyzed during the current study are not publicly available due ongoing analyses and secondary studies by the authors, but are available from the corresponding author on reasonable request.
